# Regulation of Clock-Controlled Genes in Mammals

**DOI:** 10.1371/journal.pone.0004882

**Published:** 2009-03-16

**Authors:** Katarzyna Bozek, Angela Relógio, Szymon M. Kielbasa, Markus Heine, Christof Dame, Achim Kramer, Hanspeter Herzel

**Affiliations:** 1 Max Planck Institute for Informatics, Saarbrücken, Germany; 2 Institute for Theoretical Biology, Humboldt University, Berlin, Germany; 3 Max Planck Institute for Molecular Genetics, Berlin, Germany; 4 Laboratory of Chronobiology, Charité - Universitäsmedizin Berlin, Berlin, Germany; 5 Department of Neonatology, Campus Virchow-Klinikum Charité - Universitätsmedizin Berlin, Berlin, Germany; University of British Columbia, Canada

## Abstract

The complexity of tissue- and day time-specific regulation of thousands of clock-controlled genes (CCGs) suggests that many regulatory mechanisms contribute to the transcriptional output of the circadian clock. We aim to predict these mechanisms using a large scale promoter analysis of CCGs.

Our study is based on a meta-analysis of DNA-array data from rodent tissues. We searched in the promoter regions of 2065 CCGs for highly overrepresented transcription factor binding sites. In order to compensate the relatively high GC-content of CCG promoters, a novel background model to avoid a bias towards GC-rich motifs was employed. We found that many of the transcription factors with overrepresented binding sites in CCG promoters exhibit themselves circadian rhythms. Among the predicted factors are known regulators such as CLOCK∶BMAL1, DBP, HLF, E4BP4, CREB, RORα and the recently described regulators HSF1, STAT3, SP1 and HNF-4α. As additional promising candidates of circadian transcriptional regulators PAX-4, C/EBP, EVI-1, IRF, E2F, AP-1, HIF-1 and NF-Y were identified. Moreover, GC-rich motifs (SP1, EGR, ZF5, AP-2, WT1, NRF-1) and AT-rich motifs (MEF-2, HMGIY, HNF-1, OCT-1) are significantly overrepresented in promoter regions of CCGs. Putative tissue-specific binding sites such as HNF-3 for liver, NKX2.5 for heart or Myogenin for skeletal muscle were found. The regulation of the erythropoietin (*Epo*) gene was analysed, which exhibits many binding sites for circadian regulators. We provide experimental evidence for its circadian regulated expression in the adult murine kidney. Basing on a comprehensive literature search we integrate our predictions into a regulatory network of core clock and clock-controlled genes. Our large scale analysis of the CCG promoters reveals the complexity and extensiveness of the circadian regulation in mammals. Results of this study point to connections of the circadian clock to other functional systems including metabolism, endocrine regulation and pharmacokinetics.

## Introduction

Organisms throughout evolution have developed biological clocks to better adapt to the twenty-four hour period of the solar day. Endogenous circadian oscillations have been observed in a variety of species including cyanobacteria [1], [2] and plants [3]. Circadian clocks are self-sustained oscillators that regulate the temporal organisation of physiology, metabolism and behavior [4]. In mammals, many aspects of physiology are subject to circadian regulation: sleep-wake cycles and cognitive performance, cardiac and renal functions, digestion and detoxification. About 10% of genes exhibit circadian patterns of expression in a given tissue. However, the sets of circadian regulated genes differ considerably among tissues [5], [6].

There is a hierarchical organisation of the circadian rhythm with the master clock in the suprachiasmatic nucleus (SCN) of the hypothalamus controlling peripheral oscillators in most other tissues. Light signals detected by the eyes can synchronize through the retinohypothalamic tract the phase of the SCN, but not that of the peripheral clocks. The SCN sends synchronization signals to other cells of the body putatively by hormone secretion, sympathetic enervation and indirect cues such as body temperature, feeding time and activity rhythms.

The cell-autonomous oscillations in both central and peripheral organs are generated by similar molecular components. In single cells the self-sustained oscillations are driven by interlocked transcriptional-translational feedback loops. The transcription factor heterodimer CLOCK∶BMAL1 activates the expression of *Period* genes (*Per1*, *Per2* and *Per3*), *Cryptochrome* genes (*Cry1* and *Cry2*) and nuclear receptors (*Rev-Erbα*, *Rorα*) by binding to E-box elements in their promoters. PER and CRY proteins form complexes and repress their own expression by interacting with the CLOCK∶BMAL1 dimer. REV-ERBα and RORα regulate the transcription of *Bmal1* in a separate feedback loop through ROR regulatory elements. Light input to the SCN and intercellular coupling between SCN neurons is mediated by CREB binding motifs in the promoters of clock genes such as *Per1*. Furthermore, it is known that clock output genes (e.g. *Dbp*, *Hlf*, *Tef*, *E4bp4*) regulate clock-controlled genes (CCGs) through D-boxes [7], [8]. E-boxes, ROR elements (RREs), cAMP response elements (CREs) and D-boxes are central regulatory motifs of rhythmically expressed genes [9].

The complexity of tissue- and day time-specific regulation of thousands of CCGs suggests that additional regulatory mechanisms contribute to the circadian clockwork in the central and peripheral tissues. Indeed, recent experiments show that e.g. heat-shock factor HSF1 [10], the transcription activator STAT3 [11], the transcription factor SP1 [12] or hormone receptors such as the glucocorticoid receptor (GR), thyroid receptor (TR) or estrogen receptor (ER) [13], [14] are involved in circadian gene regulation.

**Figure 1:**
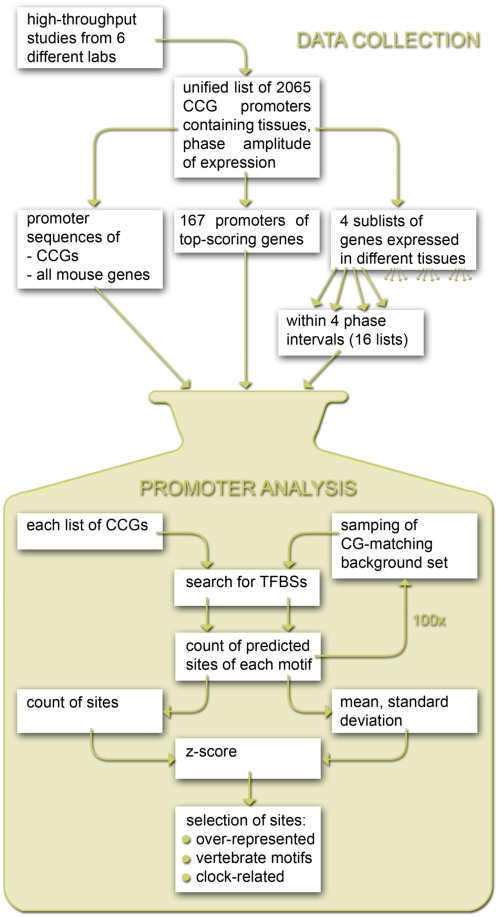
Sequential procedure of our study. The data collection consisted of a CCG study search, meta-analysis of the genes and promoter sequence collection. The meta-analysis allowed hierarchical separation of the gene list into subsets of genes expressed in 4 different tissues (heart, liver, SCN, skeletal muscle) and within each tissue into genes with the peak of circadian expression falling into 1 of 4 defined time intervals. Together with the full list of genes and a subset of genes robustly oscillating, those 22 lists were used in a TFBS overrepresentation search. The total number of predicted sites in a promoter set of interest was compared to the mean number of predictions in an iteratively sampled background promoter set (see Materials and Methods). Z-score has been used as a measure of a motif overrepresentation.

**Figure 2:**
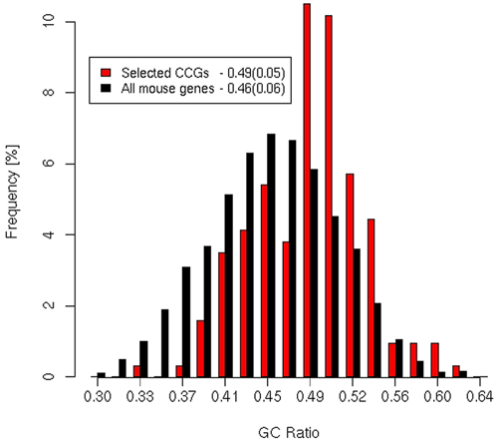
GC-content distribution of the selected subset of 167 CCG promoters versus all mouse gene promoters. Mean values of the GC-ratio and standard deviations of both distributions are indicated in the inserted box.

Our promoter analysis of CCGs is a large scale *in silico* approach to the question of regulatory mechanisms of the clock output pathways. We based our study on a meta-analysis of DNA-array data from rodent tissues. As illustrated in Figure 1 we selected six microarray studies containing complete gene annotation and full information on phases and levels of expression of genes with an oscillating circadian pattern [5], [6], [15], [16], [17], [18]. We noticed that the promoter regions of the assembled 2065 CCGs are relatively GC-rich (Figure 2). In order to avoid a bias towards GC-rich motifs we employed a novel background model. Previous promoter studies without compensation of the GC-content detected primarily GC-rich motifs [19], [20]. Using a stringent control of the false discovery rate [21] we predicted transcription factor binding sites (TFBSs) in the annotated promoter regions for all available TRANSFAC matrices. The frequencies of predicted binding sites in promoters of CCGs were compared with promoters of randomly sampled sets of mouse genes with the same GC-content which allows the use of z-scores as a measure of overrepresentation. This procedure resulted in relatively large lists of overrepresented motifs. We focus our study on transcription factors that are themselves reported as circadian expressed and on factors whose known target genes belong to our list of 2065 CCGs. By applying the analysis on lists of CCGs separated according to their tissue-specific expression, we found candidate factors involved in tissue-specific gene regulation.

## Results

### Promoter regions of clock-controlled genes are GC-rich

As described in Materials and Methods we extracted 2065 CCGs from published microarray studies. Among them we selected a subset of 167 genes that appear in at least three published gene lists, as illustrated in Figure 1. Since oscillations of these genes have been detected by independent experiments, we expect their robust circadian expression.

Previous promoter studies [22], [23], [24], [25] detected clock-related *cis*-elements several hundred base pairs upstream of the transcription start site (TSS) and also in the first intron. Accordingly, we extracted promoter regions ranging from 3 kbp upstream to 2 kbp downstream of the TSS using the EnsEmbl 43 mouse genome. Transcription factor binding sites (TFBSs) were predicted using the algorithm by Rahmann et al. [21] with a threshold of false discovery rate of 5%. The supplementary Figure S1 illustrates binding site predictions of selected transcription factors in the *Epo* gene promoter (for details see supplementary Text S1).

Mammalian promoter regions are highly heterogeneous regarding their base composition. Thus, the detection of overrepresented TFBSs requires careful consideration of the appropriate background model. In Figure 2 the GC-content of our set of selected CCG promoter regions is compared with the corresponding regions of all 25764 mouse genes available in EnsEmbl. The comparison reveals that CCGs have relatively GC-rich promoters. A naive comparison of predicted TFBSs with all mouse genes as a background would therefore lead to a bias in predictions towards GC-rich motifs such as E-boxes (consensus sequence: CACGTG) or SP1 binding sites. Consequently, we use GC-matched controls as a background model: first we determine the GC-content of the gene group of interest (as illustrated in Figure 2 for the 167 selected genes), then for each gene we sample a gene promoter from the set of all mouse genes (excluding CCGs) with the same GC-content.

**Figure 3:**
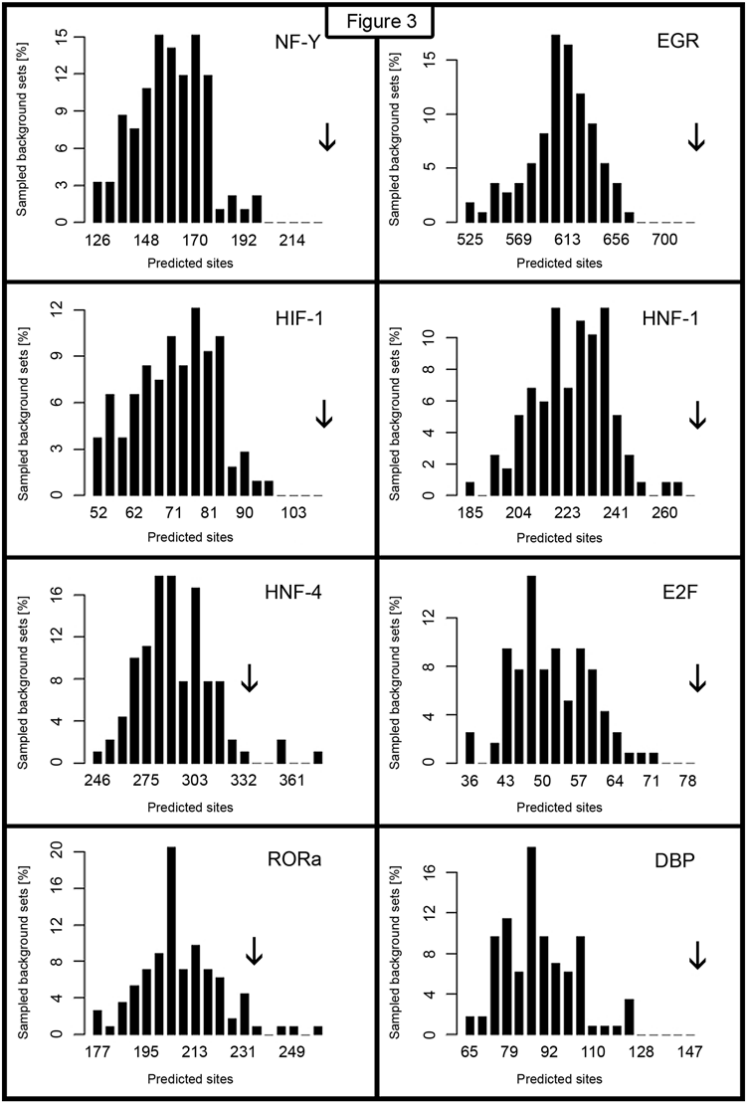
Distribution of the number of predicted TFBS. The histograms illustrate the distributions of number of predicted hits of several motifs in sampled background promoter sets with a GC-content matched to the selected subset of 167 CCGs. X-axis indicates numbers of predicted sites, the height of vertical bars corresponds to the percentage of sampled background sets containing the given number of predictions. The number of hits in the foreground set of 167 CCGs is marked with an arrow. All shown motifs are overrepresented in the analyzed CCG set (have z-scores over 2). The corresponding z-score values are given in Tables 1 and 2.

This way, the randomly obtained control gene sets had identical GC-content distribution of their promoter sequences as the analysed CCG set. We repeated this GC-matched background sampling procedure 100 times and calculated mean numbers of predicted binding sites of each transcription factor along with their standard deviations. Next, we contrasted the random sampling results with the number of predicted TFBS in the set of CCG promoters. The overrepresentation of binding sites was quantified using z-scores (Table 1). Figure 3 shows representative histograms of the number of predicted TFBSs in the background and the number of predictions in our set of 167 selected CCGs.

**Table 1:** Abundantly overrepresented *cis*-regulatory motifs in clock-controlled genes.

Motif	Consensus Sequence	Z-score in all CCGs	Overrepresentation in:				
			Sel. genes	Heart	Liver	SCN	Muscle
Sp1	DGGGYGGGVN	9.04		x	X	x	x
EGR	GYGGGSGSRRV	8.58	x	x	X	x	x
Pax-4	RNWAAWWRNNNNNNHNNNNNNNHHSAYHSB	7.06			X		x
ZF5	GSGCGCNR	6.60		x	X	x	x
AP-2	VDCCCSSVGRMS	6.35		x	x	x	x
C/EBP	NNNHKNDGNAAN	5.86	x	x	x	x	x
CRE-BP1	TTACGTAA	5.65		x	x	x	x
MEF-2	BTCTAAAAATAACYCY	5.57	x	x	x	x	x
HMGIY	HNDKNAWWTTNYYND	5.33		x	x	x	
Evi-1	DGATADGAHWRGATA	5.04	x		x	x	x
AHRHIF	NRCGTGNNN	4.92				x	x
c-Myc∶Max	VSCAYGYGSN	4.91			x		
HLF	RTTACRYMAY	4.74	x	x	x	x	x
VBP	RTTACRTMAK	4.27		x	x	x	x
E4BP4	NRTTAYGTAAYN	4.19	x	x	x	x	x
TATA	NCTATAAAAN	4.10		x	x		x
Oct-1	WNTATGBTAATT	3.82		x			
HNF-1	RGTTAATNWTTRNMN	3.67	x		x		
WT1	SVCHCCBVC	3.35					
STAT5A	NNNTTCYN	3.31					
IRF	BNNNSTTTCWNTTYY	3.30			x		
MEIS1A: HOXA9	TGACAGKTTWAYGA	3.29					
Nrf-1	CGCRTGCGCR	3.28		x			x
AhR∶Arnt	GDBNATYGCGTGMSWDBCC	3.20				x	
E2F	TTTSGCGC	3.13	x	x		x	
NF-Y	BRRCCAATVRB		6.24		x	x	x
TBP	TTTATNN		3.16	x			
HNF-4	NNNRGDBCAAAGKBCR NNN		2.80		x		
HIF-1	SNVKACGTGCNBBN		2.79			x	x
STAT1	BDNVNHTTCCSGGAAD NRNSN		2.34				
SREBP-1	NATCACGTGAB		2.09				
RORalpha1	DNWWNDAGGTCAH		2.07				
AP1	RVTGACTVMNN		2.04		x		

Motifs with a z-score above 3 in 2065 CCG promoters are shown. 8 additional factors that are overrepresented in the 167 selected gene promoters are listed below together with their z-scores from the corresponding background.

The same overrepresentation detection procedure was applied on hierarchically subdivided CCG lists. Using gene expression information we grouped genes in two different ways: according to the tissue in which the gene is circadian expressed and according to the peak time in tissue-specific circadian gene expression.

### Significantly overrepresented motifs are often clock-controlled

By combining z-scores as a measure of overrepresentation with the data on circadian expression of transcription factors and their target genes, we reduce the number of predictions as described below.

The TRANSFAC database (version 10.4) contains over 500 position weight matrices of vertebrate transcription factors. Even after clustering similar matrices [26], [27] more than 300 different vertebrate transcription factors are represented by TRANSFAC matrices. We calculated z-scores for all matrices for the following 22 gene lists: 167 selected CCGs, all 2065 CCGs, 4 tissue-specific CCG lists (mouse heart, liver, SCN, skeletal muscle) and 16 tissue- and phase-specific lists of CCGs. The tissue- and phase-specific lists were separated according to 4 time intervals that span 24 hours and are relative to the *Per2* peak of expression. Thus, each gene could be assigned to its proper expression peak bin. In order to limit the number of *in silico* predictions based on z-scores, we exploit the assembled list of 2065 CCGs. We focused our study on vertebrate transcription factors that were present in our set of CCGs and on the TFs with clock-controlled target genes, as annotated by TRANSFAC. To our surprise, many of the transcription factors with high z-scores have been reported as clock-controlled. Target genes of numerous other overrepresented transcription factors are rhythmically expressed (e.g. of EVI-1, HNF-4, MYC∶MAX, IPF1, LXR, NRF-1, GFI1, GATA-1 or NFAT). The complete results of our bioinformatic analysis are available in the supplementary Table S1 in the form of 22 lists of overrepresented and clock-related TRANSFAC matrices. The precise criteria for the matrix selection were the following:

1. Z-score of the matrix higher than 2. This threshold allows us to focus on the significantly overrepresented motifs. The lowest z-score among the known circadian-related motifs is the score of the ROR motif - 2.07. We do not observe known circadian regulatory motifs below this threshold.
2. Only the top 5% of the matrices are considered (at most 41 out of all 815 TRANSFAC matrices). This criterium shortens exceedingly long lists (all CCGs, liver).
3. We list only TRANSFAC matrices that have a direct link to our list of 2065 CCGs. This is fulfilled if a transcription factor associated with the matrix is itself a CCG or if target genes annotated in TRANSFAC are on our list of 2065 CCGs.

These criteria do not include any subjective evaluation, i.e. the compilation of the tables has been achieved automatically. The resulting tables are the major result of our study and form the basis for the further detailed analysis.

### Overrepresentation of known regulatory sites

As discussed above, E-boxes, CREB elements and ROR elements are essential motifs of the core gene regulatory network. D-boxes are considered as major elements of the clock output. It has been shown that the transcription factors DBP (VBP), HLF, TEF and E4BP4 bind to D-boxes in a phase-specific manner [8]. Indeed, the binding site motifs of E4BP4, HLF and DBP belong to the top-scoring motifs in the list of 167 selected CCGs, of all 2065 CCGs and in CCGs expressed in specific tissues (see supplementary Table S1). The TEF matrix is found in the list of CCGs expressed in the SCN with phase 0, i.e. with a similar expression peak to the *mPer2* gene (see Materials and Methods).

The RORα matrix is overrepresented in the promoters of 167 selected CCGs but with a lower z-score (Figure 3). Other nuclear receptors (GR, PR, LXR, AR, PPARγ, T3R) are found in tissue- and phase-specific lists of overrepresented motifs (Table 2).

**Table 2:** Motifs overrepresented in tissue- and phase-specific gene groups.

Tissue	Phase (genes)	Motifs
heart	all	HTF, IPF1, LXR, MZF1, USF
	0(32)	AR, CREB, E2A, LXR, MyoD, NF-κB(p65), Nrf2, PPARγ, PR(GR), STATx, T3R, USF, ZBRK1
	6(36)	GATA-3, STAT4
	12(42)	HSF2, IPF1, MZF1
	18(186)	c-Ets-1-68, NFAT, Nkx2-5, Oct-4(POU5F1), Octamer
liver	all	CLOCK∶BMAL1, HES1, NF-κB, USF
	0(384)	CREB, CREBATF, ERR-alpha, Tax/CREB
	6(298)	CLOCK∶BMAL, GFI1, HES1, Max, Nkx2-5, Stra13, USF, XBP-1
	12(403)	Cdx-2, FOXO4, GFI1B, LUN-1, NF-κB(p50), NF-κB(p65), PITX2, STAT3
	18(254)	CLOCK∶BMAL, FOXD3, FOX factors, FOXO1, FOXO4, GABP, HES1, HFH-4, HNF-3, Nkx6-2, PPARγ, USF, XFD-2
SCN	all	AhR, CREB, CREBATF, Tax/CREB
	0(85)	CREB, CREBATF, TEF
	6(78)	AhR, HSF1, LUN-1, LXR direct repeat 4
	12(137)	AhR, Pax, Tax/CREB
	18(48)	
skeletal muscle	all	AhR, Myogenin/NF-1
	0(76)	AR, HES1, HFH-4
	6(50)	GATA-4, STAT4
	12(46)	AhR, GATA-1, NF-κB, NF-κB(p50), Pbx-1
	18(114)	AhR, alpha-CP1, ICSBP

Numbers of genes in each group are indicated in parentheses. Phases are defined relative to *Per2* expression peak.

CREB elements (consensus sequence: TGACGT) are overrepresented in the list of all CCGs (with the z-score of 5.65) and appear as overrepresented in all tissue-specific motif lists (Table 1). Hits of the CLOCK∶BMAL1 matrix are overrepresented in the promoters of liver-specific CCGs (z-score: 3.08). Several other E-box-like motifs such as c-MYC∶MAX, USF, STRA13 (DEC1), MyoD or SREBP-1 are overrepresented as well. Additionally, the recently discovered circadian regulators STAT3 [11], HSF1 [10], SP1 [12] and nuclear factors (LXR/RXR, GR, ERRα, PPARγ, HNF-4, T3R) [13], [28] occur in several lists of overrepresented sites. These examples support the conclusion that our bioinformatic analysis identifies known clock-related regulatory elements as overrepresented in promoter regions of CCGs.

### Competition of multiple transcription factors for E-boxes, D-boxes and ROR elements

Redundancy among transcription factor binding sites has been extensively discussed [26], [27], [29], [30]. For example, many bHLH transcription factors bind to E-boxes. Consequently, we find in our lists in addition to CLOCK∶BMAL1 several other E-box binding factors (supplementary Figure S2). Interestingly, almost all of them belong to the set of 2065 clock-controlled genes. It is likely that some of these factors compete for binding at functional E-boxes as has been shown for D-box binding transcription factors [8]. In order to quantify possible competition for E-boxes, expression levels and affinities of the relevant factors need to be determined quantitatively.

**Figure 4:**
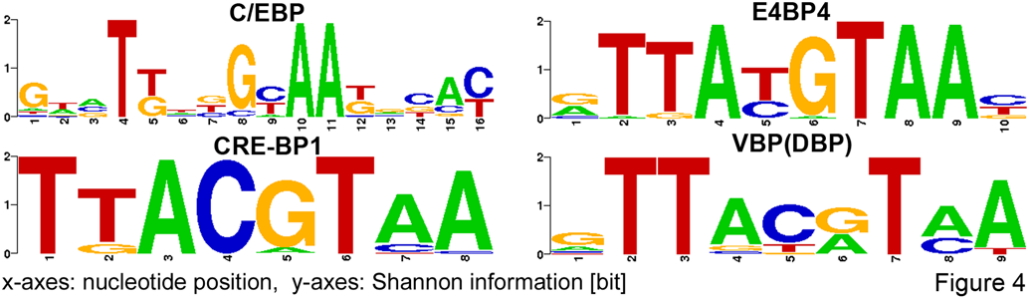
Sequence logos of binding sites showing strong similarity to a D-box. Both D-boxes and E-boxes (supplementary Figure S2) are known to regulate clock genes and clock output pathways. Motifs highly similar to D-boxes and E-boxes are found to be overrepresented in the promoters of CCGs.

The D-box binding bZIP transcription factors, DBP (VBP), HLF, TEF and E4BP4, have the consensus sequence TTAYRTAA (where Y is a pyrimidine and R a purine). Figure 4 shows that position weight matrices of other transcription factors, such as CRE-BP1 or C/EBP show similarities to D-boxes. The 5 most conserved positions of the C/EBP motif represent the consensus sequence. This observation points to a limitation of our search for overrepresented sites: the high frequency of D-boxes in CCGs promoters might lead to false positive overrepresentations of other D-box-similar binding sites. On the other hand, C/EBP has been reported to be circadian regulated in liver and heart, and thus a competition with known D-box regulators is possible. Moreover, known target genes of C/EBPβ (*Cyp7a*, *Otc*, *Ttr*, *Adh1*, *Slc2a2*, *Sst*, *Orm1*, *Cdkn1a*, *C/Ebpα*, *Icam1*, *Top1*) and of CRE-BP1 (*Fn1*, *Plat*, *Spp1*, *Ifn-β*) have been also reported as clock-controlled [5], [6], [16], [17], [18].

Since hormone response elements resemble each other, competing regulation of clock-controlled genes is likely. A well documented example is the antagonistic binding of REV-ERBα and RORα to the *mBmal1* promoter [31].

### Prediction of novel regulatory motifs

In Table 1 we present a list of transcription factors with overrepresented binding sites in the promoter regions of clock-controlled genes. As discussed above, many TFs of the overrepresented motifs (17 out of 34) are CCGs themselves. Several of the high-scoring motifs are built up of GC-rich binding sites. Since we apply a GC-matched background model, this result may not be biased in the promoter DNA composition. The analysis by Reinke and others [10] detected differential circadian binding of proteins to DNA elements rich in GC. Some of the predicted factors are also AT-rich (MEF-2, HMGIY, OCT-1, HNF-1, TBP).

### Tissue- and phase-specific transcription factors

So far, we analyzed clock-related transcription factors with overrepresented binding sites in the list of all CCGs. These factors appear again in many lists of tissue-specific genes (see supplementary Table S1). In Table 2 we present additional transcription factors with motifs overrepresented solely in given tissue- and phase-specific lists. These transcription factors might contribute to the fine-tuning of the circadian clockwork in peripheral tissues.

The listed factors include the nuclear receptors AR, LXR, PPARγ , PR(GR) and ERRα. We find several regulators of the immune response as well (STAT4, GATA-3, NF-κB), in particular in the liver. Motifs of the myocyte enhancer factor MEF-2 can be found with high z-scores (Table 1). In the lists referring to heart and muscle tissues additional muscle-specific factors appear, such as E2A, NKX2-5, MyoD and Myogenin/NF-1.

CRE-binding factors are overrepresented in the SCN, reflecting the light input pathways and coupling via neurotransmitters [28]. The liver-specific transcription factors HNF-1, HNF-3, HNF-4 and C/EBP [32] are found to be overrepresented in CCG promoters from liver experiments

### *Epo* gene expression in the adult kidney underlies circadian oscillation

**Figure 5:**
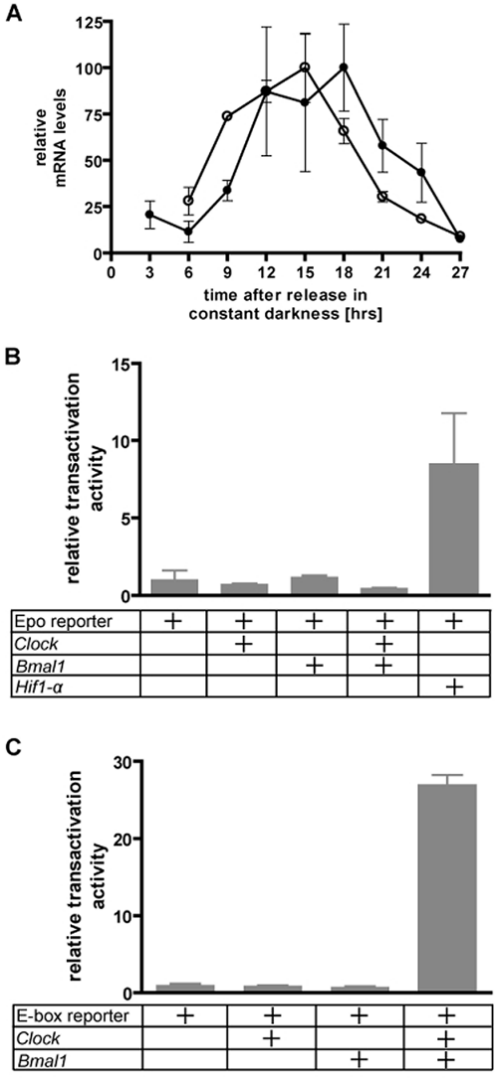
Analysis of circadian regulation of *Epo*. (A) Quantitative PCR analysis of circadian *Epo* and *Per2* mRNA expression in the adult murine kidney over a time period of 24 hours after release in constant darkness and normoxia. *Epo* mRNA (filled circles) and Per2 (open circles) mRNA transcript levels were normalized to Gapdh mRNA levels. Values are given as means±SD. (B) Analysis of the activity of a reporter gene construct harboring the 5′ promoter, first intron and 3′ enhancer of the human *Epo* gene (upper panel) or an E-Box reporter construct (lower panel) both co-transfected with Clock or Bmal1 alone or combined in human neuronal precursor (SH-SY5Y) cells. As positive control for the *Epo* reporter activation cells were also co-transfected with a HIF-1α expression plasmid (upper panel). A renilla luciferase vector was used for the normalization of transfection efficiencies. Values are shown as means±SD of three independent experiments, each performed in duplicate.

Based on the finding of several predicted binding sites within the *Epo* promoter region for transcription factors that are regulated in circadian rhythms (supplementary Figure S1), we quantitatively analysed circadian *Epo* and *Per2* mRNA expression in the adult kidney. During a 24-hour period after release in constant darkness under normoxia, *Epo* mRNA expression increased up to nine-fold with a peak between CT12 to CT18 corresponding to the first half of the subjective night. Analysis of *Per2* mRNA expression confirmed intact endogenous clockwork activity (Figure 5A).

Since the 5′ minimal *Epo* promoter contains an E-box motif, we tested whether the transcription factors CLOCK and BMAL1 activate the *Epo* reporter gene construct. Efficacy of heterodimeric CLOCK and BMAL1 activity was confirmed in the E-box reporter assay (Figure 5C). CLOCK and BMAL1 had no effect on the *Epo* activity, while HIF-1α, activated the reporter gene construct about 10-fold (Figure 5B).

## Discussion

Our aim was to predict regulatory mechanisms of the mammalian circadian clockwork by a large scale promoter analysis of clock controlled genes (CCGs). We found that promoters of CCGs reported in several DNA-array studies exhibit relatively high GC-content. Consequently, our analysis is based on GC-matched control promoters chosen randomly from the mouse genome. This way we obtained z-scores quantifying the overrepresentation of transcription factor binding sites (TFBSs) in promoters of CCGs.

### Confirmation of known regulatory sites

Among our predictions we found known regulatory sites such as E-boxes, D-boxes, CREB elements and hormone response elements. D-boxes obtain particularly high scores pointing to a central role of the transcription factors DBP, HLF and E4BP4. Nuclear receptor binding sites of HNF-4, RORα, AR, LXR, PPARγ, GR, T3R and ERRα were found predominantly in tissue-specific analysis, which is in agreement with the nuclear expression atlas [13]. CREB binding sites are significantly overrepresented in SCN-specific prediction lists, presumably due to its dependency on the light input.

### Prediction of novel transcriptional regulators

For a detailed discussion we exploit the list of 2065 CCGs. Many of the novel predicted regulators appear on the list of 2065 CCGs others have annotated target genes included in the list (e.g. SP1, AP-2, NF-Y). These transcription factors are compiled in Tables 1 and 2. Complete lists of significantly overrepresented transcription factor binding sites are provided in the supplementary Table S1.

How reliable are these predictions? Due to short length and low information content of binding site motifs, prediction of individual TFBSs is often error-prone, leading to many false positives [33]. However, our study is based on a combined analysis of large sets of CCG promoter regions compared with GC-matched controls. This approach allows a quantification of the overrepresentation by the means of z-scores in spite of potential false positive predictions. Remarkably, all known regulators are among our predictions. The REV-ERBα matrix is not included in the TRANSFAC database (version 10.4), nevertheless its closely related nuclear receptor RORα is overrepresented. Moreover, the recently described additional regulators HSF [10], STAT3 [11], SP1 [12] as well as PPARγ, GR, ERRα, RXR, TR, SREBP-1 [13] belong to our predictions. Tables 1 and 2 represent about 22% of all vertebrate binding motifs in TRANSFAC (version 10.4), and the fact that essentially all known circadian regulators are in the tables strongly supports our bioinformatic approach.

In the following we show that many of the predicted transcription factors play indeed essential roles in hormonal, metabolic and detoxification regulatory networks.

### Endocrine regulation

It is well established that hormones such as glucocorticoids [34], vasopressin [35], adrenocorticotropic hormone (ACTH) [36], [37] and thyrotropin [38], [39] have pronounced circadian rhythms. Moreover, several hormone receptors and the serum glucocorticoid kinase 1 (SGK1) belong to our list of clock-controlled genes. Some of our predicted transcription factors are closely related to hormone regulation. Supplementary Figure S3 shows a functional classification of the predicted factors, for its description see supplementary Text S2.

**Figure 6:**
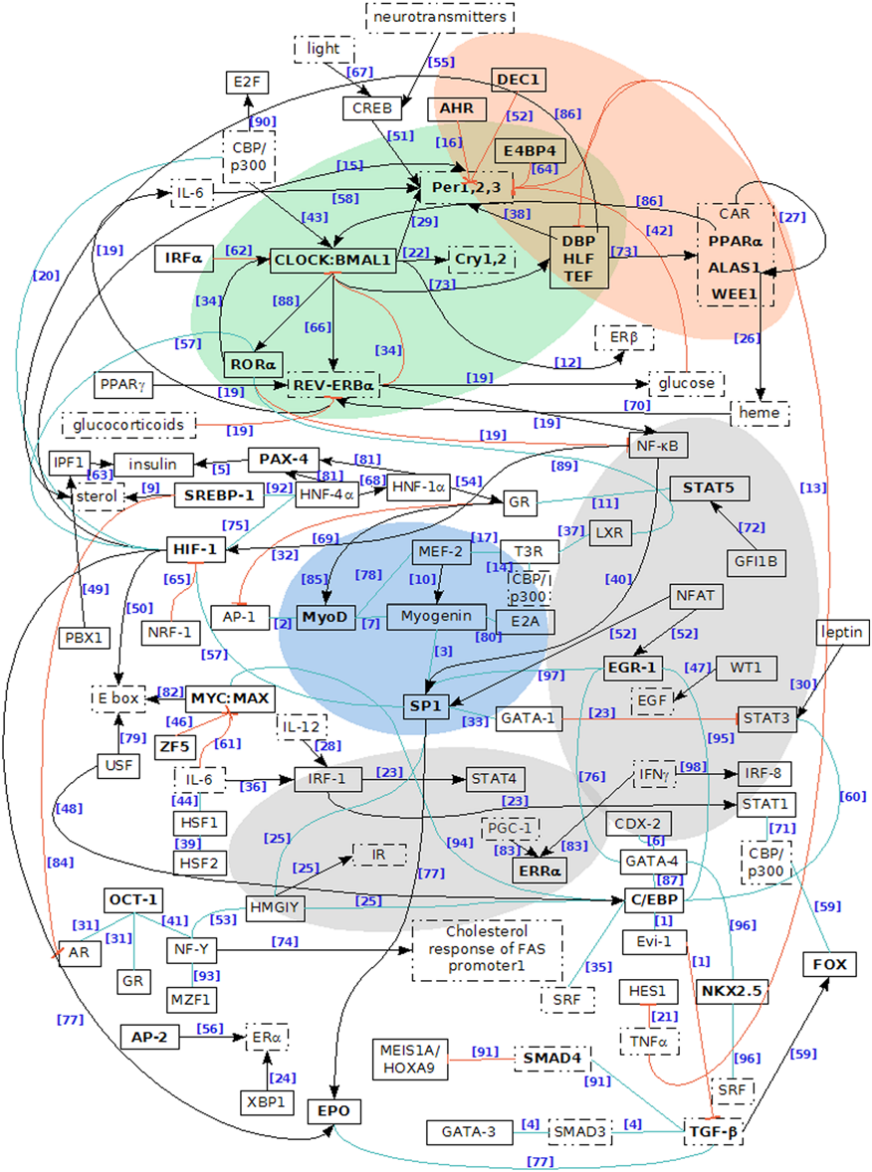
A putative network of circadian regulation. The figure illustrates our computational predictions nested in other regulatory interactions reported in the literature. Solid line boxes contain transcription factors predicted by our study to regulate clock controlled genes. Dashed line boxes contain other factors involved in the regulation of clock controlled genes as provided by the literature. Transcription factors in bold letters are those reported to be clock controlled by at least one of the microarray studies mentioned in the main text or in other publications [58], [59]. Black arrows indicate activation, red dead-end lines inhibition and blue simple lines represent interaction. References to the literature reporting on particular interactions are indicated next to each connecting line. The full reference list can be found in the supplementary material. Several functional groups are highlighted: core clock proteins in green, proteins related to metabolism and detoxification in red, immune system related proteins in grey and muscle-specific in blue.

Even though hormone receptor recognition motifs do not display the highest z-scores, the combined action with other overrepresented transcription factor motifs might play a role in the regulation of many clock-controlled genes. Tronche and others mention the competition of the GR with AP-1, NF-κB, CREB, GATA-1 and OCT-1 as well as its interaction with C/EBPβ or STAT5 in transcriptional regulation [37]. These interactions are shown as part of a large network (Figure 6) involving also others CCGs and core clock genes. The network provides an in-depth analysis of the predicted factors and their interactions with the different functional groups. References supporting the figure are provided in the supplementary Text S3.

### Transcriptional regulation of Erythropoietin (*Epo*)

Our experimental data provides evidence of circadian oscillation of *Epo* gene expression in the kidney (Figure 5), which is the primary site of *Epo* production in adults [40], [41], [42]. This is a noteworthy finding, since diurnal changes in circulating *Epo* concentrations (both at sea level and at high altitude) have been previously reported in humans [43], [44], [45].

The murine and human *Epo* promoter regions contain several potential binding sites for transcription factors (supplementary Figure S1), whose functional implication has not been completely elucidated yet. Since the minimal *Epo* promoter contains an E-box motif, which could represent a binding element for the transcription factor heterodimer CLOCK∶BMAL1 as the major circadian regulator, we tested herein the hypothesis that these factors could be involved in diurnal oscillation of *Epo* mRNA expression. Our reporter gene analysis indicated that CLOCK and BMAL1 do not play a direct role in regulating *Epo* promoter activity (Figure 5). We show that one of our predicted transcription factors, HIF-1α, activates the *Epo* reporter gene construct increasing its expression by a factor of 10.

### Circadian regulation of metabolism

Our lists of overrepresented binding sites include many circadian regulators of metabolism. Tables 1 and 2 contain the PAR bZip proteins HLF, DBP(VBP), E4BP4 and TEF, the nuclear receptors HNF-4, RORα, LXR, PPARγ, PR(GR) and ERRα, as well as common regulators such as C/EBP, SREBP-1 and HIF-1. Moreover, some additional transcription factors with links to the energy metabolism are among our predictions: EVI-1 as an inhibitor of C/EBPα [46], NRF-1 as an inducer of aminolevulinic acid (ALA) synthase expression [47], IPF1 as a regulator of insulin expression [48], and NF-Y as a coactivator of cholesterol response [49].

In our putative network of circadian regulation (Figure 6) we embedded the predicted regulatory proteins as well as other factors and regulatory relations reported in the literature. The section of the network highlighted in red contains proteins related to metabolism.

### Chronopharmacokinetics

Absorption, distribution, metabolism and elimination of drugs are subject to pronounced diurnal fluctuations [50]. The D-box binding transcription factors HLF, DBP and TEF regulate major detoxifying enzymes (ALAS1, POR) and nuclear receptors (CAR, PPARs) [7]. Among the transcription factors known to be involved in xenobiotic detoxification [50] most are overrepresented in our prediction lists: AHR, HLF, DBP, TEF, HTF, HNF (Tables 1 and 2). Furthermore, HSF1 has been discussed in the context of detoxification [51], whereas NFR-1, predicted in this study, is important for the induction of antioxidant enzymes [52]. The overrepresented binding sites of MYC∶MAX (Table 1) might be relevant for the cross-talk between cell cycle, circadian clock and chemotherapy [53].

### Chronoimmunology

Several of the overrepresented transcription factors are involved in the immune response (Tables 1 and 2, supplementary Figure S3 and Text S2). The network of transcription associated with the immune system is highlighted in grey in Figure 6. As seen in the figure, not only the clock influences the immune system but also the immune system can feed back to the clock, as demonstrated by the negative action of TNF on core clock components. These feedbacks strengthen the idea of an interconnected network ruled by the clock mechanism.

### Hierarchical transcription factor network

Our approach, based on a bioinformatic analysis of CCG promoters, does not allow to distinguish core regulatory elements (E-boxes, ROR elements), input pathways (CREB elements), direct output (D-boxes) and secondary regulation (via hormones, nervous system, feeding, sleep-wake cycle or body temperature). Most of the DNA-arrays used in our meta-analysis measured expression profiles in constant darkness, therefore, we do not expect major regulation effects from light input. The described set of overrepresented transcription factors forms a large network with strong links to the endocrine system, metabolism and the immune system (Figure 6). Some of our computational predictions are supported by recent experimental studies (e.g. HSF1, SP1, STAT3, SREBP-1).

Our approach cannot directly address the important role of co-factors such as PGC1-α [54] or CBP/p300 [55] in the transcriptional regulation of CCGs. We included these regulators, as well as other essential factors such as steroids, IL-6, TMPα, heme, insulin, leptin, ALAS1, in dashed boxes in the graphical summary of our predictions (Figure 6). The figure illustrates how the computational predictions (solid line boxes) are embedded in a large regulatory network of clock-controlled genes. It contains interconnections between different biological functional components as well as their interdependency with the core clock. The network presents in a concise way complex regulatory relations of circadian clock in mammals. It affords therefore a broad source of reference for further focused study and represents the main result of our analysis.

## Materials and Methods

From published microarray analyses detecting genes with circadian expression we selected 6 studies on mammalian tissues and containing information on gene expression phases and amplitudes [5], [6], [15], [16], [17], [18]. Through the unification of the overlapping genes and removal of annotation inconsistencies a list of 2065 different transcripts of clock controlled genes has been assembled containing genes expressed in rat fibroblasts and mouse tissues, such as liver, heart, SCN and skeletal muscle. The meta-analysis of the genes revealed a limited overlap among the tissues: only 77 genes are expressed in 2 tissues, 23 in 3 and more. The mean deviation of measured peak expression phases of the same gene expressed in two different tissues (e.g. 5.8 h for the liver-SCN genes, 4.5 h for liver-heart genes) is consistent with the observations from other studies [5].

From the complete list of the CCGs we selected a subset of 167 promoters of top-scoring genes that have been reported in at least three published experiments. The fact that these genes have been detected by independent experiments indicates their robust circadian expression. Using the gathered expression information we performed the promoter analysis on groups of CCGs organized in a hierarchical manner. The initial complete list was first subdivided into tissue-specific gene sets that where then regrouped into sets of genes having the same expression phase within each tissue. The phases were calculated relative to the *Per2* expression phase and grouped into 4 time intervals each of the length of 6 hours.

We extracted sequences ranging over 3 kbp upstream and 2 kbp downstream of the transcription start site (TSS) of each gene. The choice of the region was motivated by previous promoter studies [22], [23], [24], [25], [56] detecting clock-related *cis*-elements within several hundred base pairs upstream of a gene TSS as well as on its first intron. The sequences have been downloaded from EnsEMBL 43 mouse genome. We used the method of Rahmann [21] for the background model computation and the cut-off threshold estimation in the prediction of transcription factor binding sites. The implementation of the algorithm is a part of the BioMinerva framework (SM Kielbasa, in preparation), the chosen threshold of false discovery rate is the default 5%.

The promoter regions were scanned for overrepresentation of putative cis-elements as compared with a background set of genes not known to be oscillating. All 815 position specific count matrices from the TRANSFAC version 10.4 were used and the search of the binding sites was performed on the 5 kbp promoter regions of each analyzed CCG subset. The number of predicted sites of each motif was calculated and compared to the corresponding average number of predicted sites in sampled background promoter sets. The background promoter set is sampled 100 times randomly from promoters of all EnsEMBL mouse genes excluding those reported as clock controlled. Additionally, during the sampling procedure the GC-content of the analysed sequence group is considered. We define GC-content intervals of the width of 1% and calculate the number of genes in the analysed set falling into each of such GC-content bins. The proper number of background sequences of each of the CG-content bins is sampled. This background model was motivated by the observation that the CCG promoters tend to have a higher GC-content as compared to other mouse gene promoters (Figure 2). Using such a GC-matched background model assures that the overrepresentation of certain motifs is not due to the bias in the sequence nucleotide composition.

For further analysis we selected only vertebrate motifs having the number of predicted binding sites at least 2 standard deviations above the mean number of its predicted binding sites in the sampled reference promoter set. Assuming a Gaussian distribution of the number of predictions (compare Figure 3), a z-score above 2 implies about 2.3% false positives. We found that out of 100 sampled control sets about 2 sets exceed the threshold of two standard deviations (data not shown). Moreover, we used the TRANSFAC database to select from all overrepresented motifs those, whose transcription factors appeared on the CCG list and those that regulate oscillating target genes. Genes fulfilling all the above criteria are considered as the final result of this analysis.

Firstly, this overrepresentation search procedure was performed on the promoters of both all CCGs and of the selected subset of robustly oscillating genes. Next, we subdivided the full list into tissue-specific gene lists. The liver gene list is strikingly larger – over 3 times than other tissues lists, presumably due to the prevalence of liver experiments or to the fact that circadian regulation in liver is particularly strong and widespread. Finally, each tissue-specific list has been subdivided into 4 lists of genes with the peak of expression belonging to a proper phase interval.

### Analysis of circadian *Epo* gene expression in the murine adult kidney

*Epo* mRNA expression levels were analyzed by real-time PCR. Adult murine kidney specimens (C57BL/6 mice) were obtained at defined time points (every 3 hours; n = 2 to 4, each) after release of entrained mice in constant darkness. Total RNA was prepared by using a commercial kit (Qiagen, Hilden, Germany), and used as a substrate for cDNA synthesis and quantitative real-time PCR using the reagents and instructions of the PCR machine manufacturer (Applied Biosystems, Foster City/CA, USA). The following primer and probe sequences were used for PCR amplification: GAPDH: FW 5′- TGT GTC CGT CGT GGA TCT GA -3′; RW 5′- CCT GCT TCA CCA CCT TCT TGA -3′; probe: FAM- CCG CCT GGA GAA ACC TGC CAA GTA TG - TAMRA; Per2: FW 5′- ATG CTC GCC ATT CAC AAG A -3′; RW 5′- GCG GAA TCG AAT GGG AGA AT -3′; probe: FAM- ATC CTA CAG GCC GGT GGA CAG CC -TAMRA. For *Epo* expression analysis a commercial Taqman Gene Expression Assay (Mm00433126m1; Applied Biosystems) was used. Transcript levels were compared on the basis of differences in the threshold cycles (C_t_) values. Only samples with equal levels of GAPDH mRNA (±0.5 C_t_) were taken into account. Transcript levels of the genes of interest are normalized to those of GAPDH.

### Cell transfection and reporter gene assays

Human neuroblastoma-derived cells (SH-SY5Y; ATCC No. CRL-2266), an established cell line for analyzing *Epo* gene regulation in neuronal cells with a fetal phenotype, were grown in DMEM, 10% fetal calf serum, and 1∶100 P/S to a confluence of approximately 60 to 70%. After 24 hours, cells were transfected with the following plasmids using Lipofectamine™\ 2000 transfection reagent (Invitrogen, Karlsruhe, Germany) at a 4∶1 (Lipofectamine™∶DNA) volume ratio : pGL2 basic vector (control) or pGL2p117Le126 (kindly provided by Kerry L. Blanchard) that harbors the 5′ 117-bp *Epo* promoter sequence, the first intron, and the 3′ 126 bp *Epo* enhancer fragment with the hypoxia response element (NCBI accession no. M11319.1. Nucleotide (nt) 270 to 661 plus nt 3449 to 3575). Effects of CLOCK and BMAL1 were analyzed by co-transfection of vector plasmids: pDEST26-clock and pDEST26-bmal1 300 ng each. The HIF-1α expression plasmid (pcDNA3hHIF1α, kindly provided by Eric Metzen, University of Essen) served as positive control for the induction of the pGL2-p117e126-L-Epo reporter. The luciferase reporter vector pGL3_E6S containing 6 E-boxes of the *mPer1* promoter served as positive control for the efficacy of CLOCK and BMAL1 transfection [57]. Cells were additionally transfected with vector plasmid, coding for renilla luciferase (phRL-SV40) as a control for transfection efficacy. 48 hours after transfection, cells were harvested and analyzed for luciferase activity using the Dual-Luciferase Reporter Assay System protocol (Promega, Madison/WI, USA). Results are shown as averages of three transfection experiments, each performed in triplicate.

## Supporting Information

Figure S1 Murine erythropoietin (Epo) promoter. The figure shows a set of selected transcription factor binding sites predicted with a false discovery rate of 5% [7] and the corresponding p-values.
(6.96 MB TIF)

Figure S2 Competition of E-box binding sites. Sequence logos of binding sites showing strong similarity to E-box. E-box are known to regulate clock genes and clock output pathways. Motifs highly similar to E-box (consensus sequence: CACGTG) are found to be overrepresented in the promoters of CCGs.
(5.90 MB TIF)

Figure S3 Functional classification of overrepresented transcription factors. The predicted factors are assigned to organ-specific or functional systems.
(0.72 MB TIF)

Text S1 Regulation of Epo gene.
(0.04 MB DOC)

Text S2 Functional classification of overrepresented transcription factors.
(0.07 MB DOC)

Text S3 Additional references to the regulatory network presented in Figure 6.
(0.08 MB DOC)

Table S1 Overrepresented motifs in all analysed gene lists.
(0.09 MB XLS)
