# Supplementary figures and images for: Regulation of Clock-Controlled Genes in Mammals
Source: PLoS One. 2009 Mar 16;4(3):e4882. doi: 10.1371/journal.pone.0004882 (PMC2654074; doi:10.1371/journal.pone.0004882)

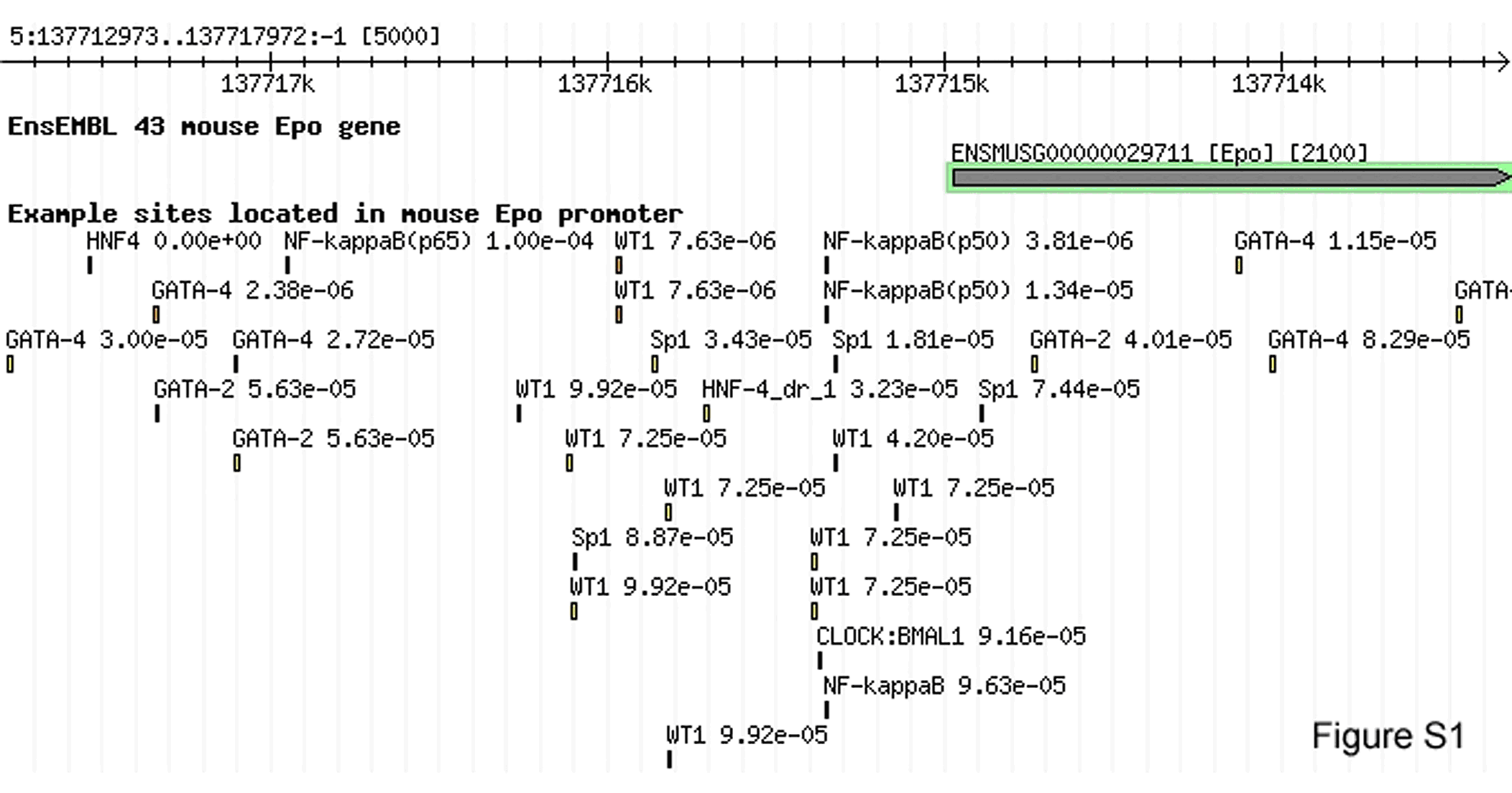

Supplement: Figure S1 — Murine erythropoietin (Epo) promoter. The figure shows a set of selected transcription factor binding sites predicted with a false discovery rate of 5% [7] and the corresponding p-values. (6.96 MB TIF) [file pone.0004882.s001.tif]

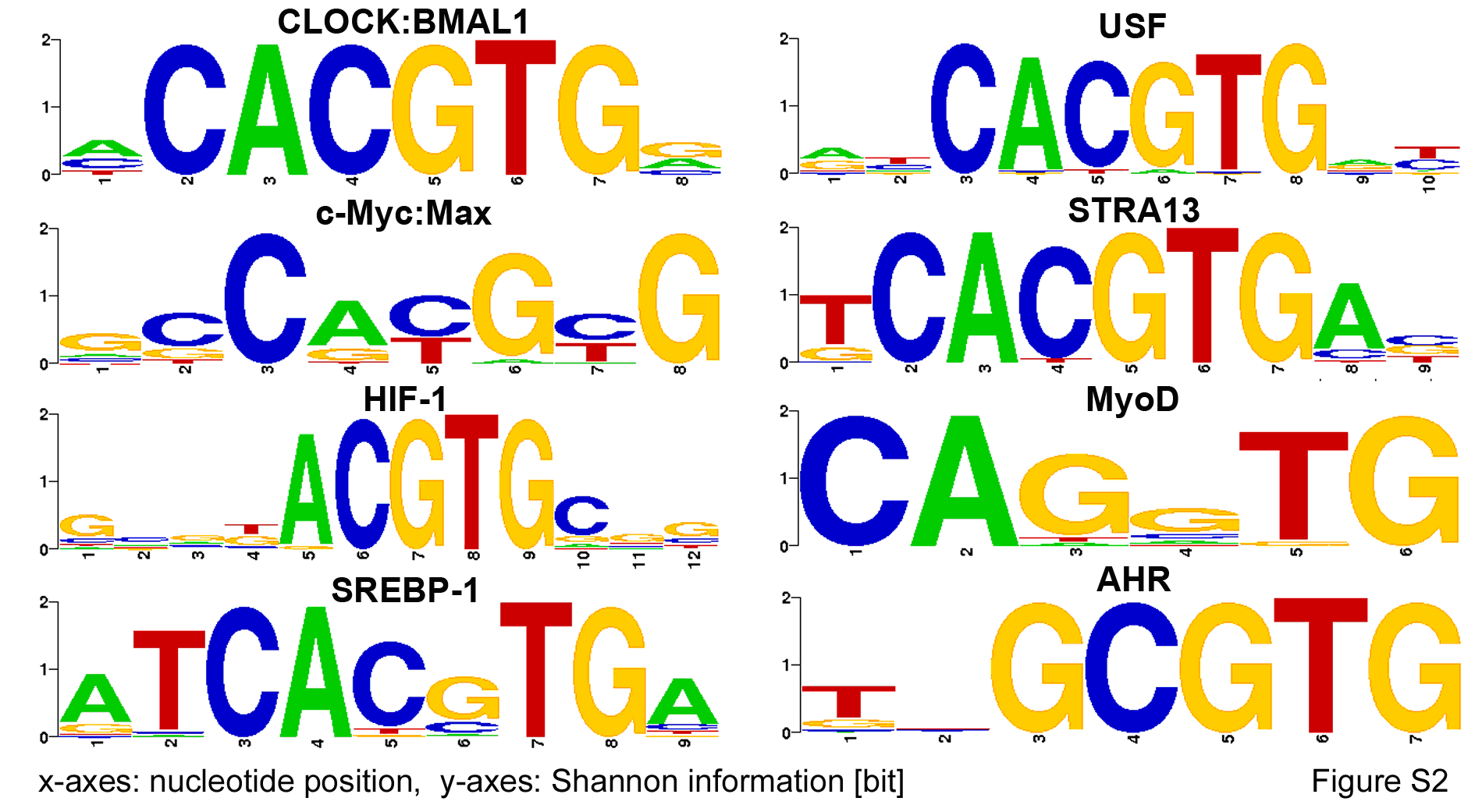

Supplement: Figure S2 — Competition of E-box binding sites. Sequence logos of binding sites showing strong similarity to E-box. E-box are known to regulate clock genes and clock output pathways. Motifs highly similar to E-box (consensus sequence: CACGTG) are found to be overrepresented in the promoters of CCGs. (5.90 MB TIF) [file pone.0004882.s002.tif]

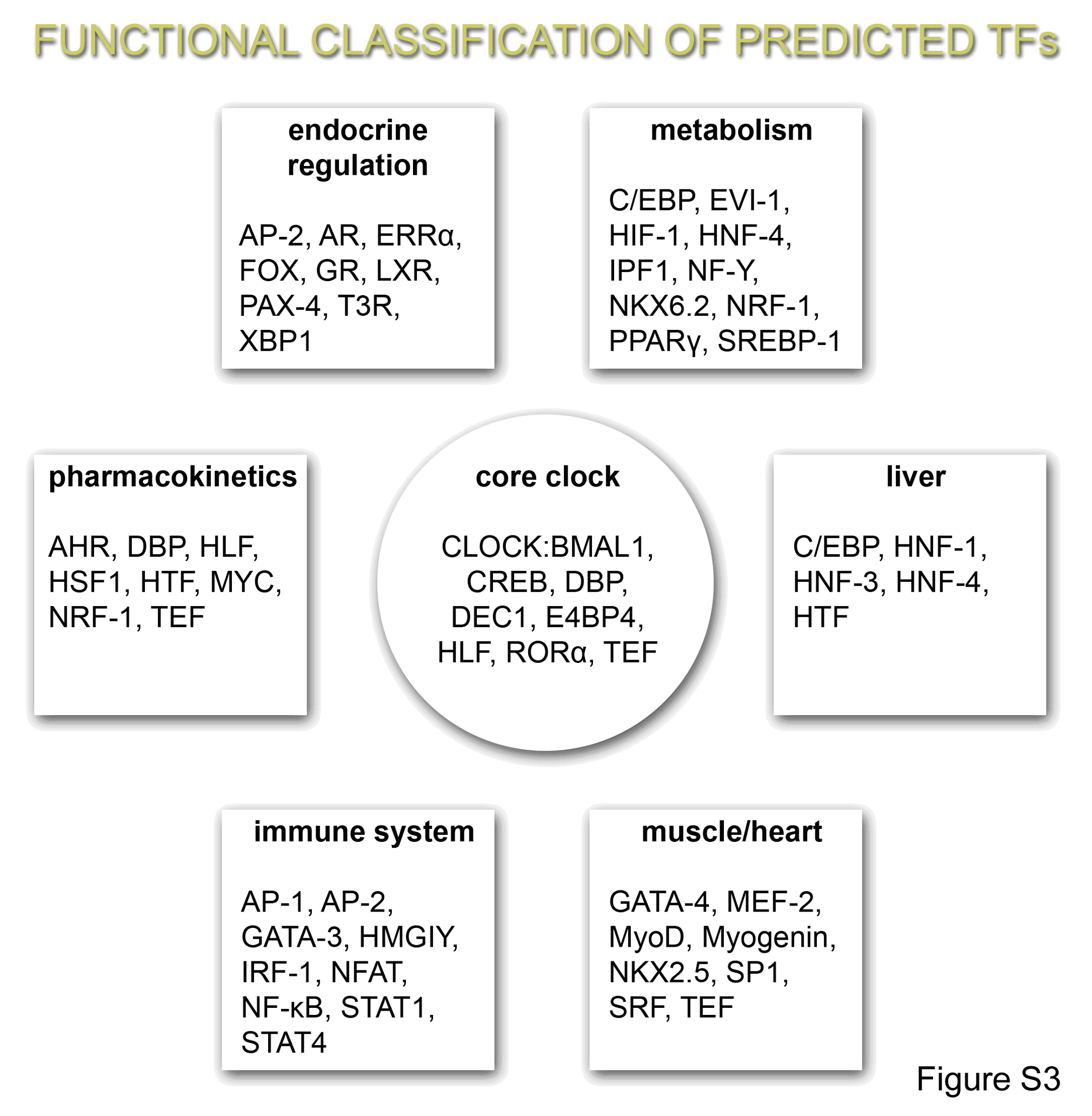

Supplement: Figure S3 — Functional classification of overrepresented transcription factors. The predicted factors are assigned to organ-specific or functional systems. (0.72 MB TIF) [file pone.0004882.s003.tif]
