# Supplementary material for: Regulation of Clock-Controlled Genes in Mammals
Source: PLoS One. 2009 Mar 16;4(3):e4882. doi: 10.1371/journal.pone.0004882 (PMC2654074; doi:10.1371/journal.pone.0004882)
Supplement: Text S1 — Regulation of Epo gene. (0.04 MB DOC) [file pone.0004882.s004.doc]

**Supplemental Text S1: Regulation of *Epo* gene**

Regulation of *Epo* gene expression is complex, since *Epo* is not only regulated in response to hypoxia [1]. Besides the adult kidneys as a primary production site for *Epo*, the *Epo* gene is also expressed in various other organs such as (fetal) liver, brain, and reproductive organs [2,3,4]. Cumulative data provide evidence for the developmental stage- and tissue-specific regulation of *Epo*, both under normoxia and hypoxia [1]. In hepatocytes of the fetal liver, which is the primary site of *Epo* production during development [4,5], and in human hepatoma (Hep3B, HepG2) cells, which exhibit a fetal phenotype and are well established for *in vitro* analysis of *Epo* regulation, *Epo* promoter activity is regulated by SP1, WT1 and GATA-4 [6,7,8]. As indicated in our analysis, these factors bind to DNA motifs that are overrepresented in CCGs (Table 1). Tissue-specific transcriptional activators of renal *Epo* expression have not been identified yet. However, non-hypoxic renal *Epo* regulation may involve repressive activities binding to the promoter element [6]. In that context, the identification of regulatory DNA-motifs that are overrepresented in clock-controlled genes and specifically expressed in the kidney may direct future studies on mechanisms of circadian *Epo* gene oscillation.

**References**

1. Fandrey J (2004) Oxygen-dependent and tissue-specific regulation of erythropoietin gene expression. Am J Physiol Regul Integr Comp Physiol 286: R977-988.

2. Chikuma M, Masuda S, Kobayashi T, Nagao M, Sasaki R (2000) Tissue-specific regulation of erythropoietin production in the murine kidney, brain, and uterus. Am J Physiol Endocrinol Metab 279: E1242-1248.

3. Dame C, Bartmann P, Wolber E, Fahnenstich H, Hofmann D, et al. (2000) Erythropoietin gene expression in different areas of the developing human central nervous system. Brain Res Dev Brain Res 125: 69-74.

4. Dame C, Fahnenstich H, Freitag P, Hofmann D, Abdul-Nour T, et al. (1998) Erythropoietin mRNA expression in human fetal and neonatal tissue. Blood 92: 3218-3225.

5. Zanjani ED, Poster J, Burlington H, Mann LI, Wasserman LR (1977) Liver as the primary site of erythropoietin formation in the fetus. J Lab Clin Med 89: 640-644.

6. Obara N, Suzuki N, Kim K, Nagasawa T, Imagawa S, et al. (2008) Repression via the GATA box is essential for tissue-specific erythropoietin gene expression. Blood 111: 5223-5232.

7. Rahmann S, Möller T, Vingron M (2003) On the Power of Proﬁles for Transcription Factor Binding Site Detection. Stat Appl Genet Mol Biol 22: Article7.
