# Supplementary material for: Regulation of Clock-Controlled Genes in Mammals
Source: PLoS One. 2009 Mar 16;4(3):e4882. doi: 10.1371/journal.pone.0004882 (PMC2654074; doi:10.1371/journal.pone.0004882)
Supplement: Text S2 — Functional classification of overrepresented transcription factors. (0.07 MB DOC) [file pone.0004882.s005.doc]

**Supplemental Text S3: Functional classification of overrepresented transcription factors.**

Among factors involved in hormone regulation we identify PAX-4, a protein involved in the differentiation of endocrine progenitors [1,2] to islet hormone promoters [3]. The clock-controlled gene XBP1 enhances the activity of the estrogen receptor ERα [4]. Also the predicted FOX factors, as well as AP-2, are known to regulate ERα [5,6,7]. Furthermore, a strong synergy is found between the circadian clock and energy metabolism [8,9]. On the one hand, D-box binding transcription factors [10], nuclear receptors (e.g. REV-ERBα, RORα) and metabolic hormones induce alterations between catabolic and anabolic states [11]. On the other hand, feeding can entrain circadian rhythms [12] even in the absence of the master clock in the SCN [13]. A comprehensive expression analysis of all 49 mouse nuclear receptors (NRs) revealed that 28 of them are expressed with circadian rhythms in fat, liver and muscle tissues [14]. Recent reviews discuss a role of the transcription factors HNF-4α, C/EBP, SREBP-1 and HIF-1 as circadian regulators [9,17]. The liver-specific transcription factors HNF-1, HNF-3, HNF-4 and C/EBP [15] are found to be overrepresented in CCG promoters from liver experiments. Muscle-specific factors [16], such as MEF-2, MyoD, Myogenin, E2A and SP1 are predicted in heart and muscle tissue. Key components of the immune system exhibit as well circadian variations. For example, the effects of lipopolysaccharides (LPS) vary in the course of the day [18], B cell survival is modulated by the circadian clock [19] and STAT3 signaling shows circadian rhythm [20]. Mediators such as TNFα and IL-6 are subject to distinct diurnal rhythms [9].

**References**

1. Smith SB, Ee HC, Conners JR, German MS (1999) Paired-homeodomain transcription factor PAX4 acts as a transcriptional repressor in early pancreatic development. Mol Cell Biol 19: 8272-8280.

2. Sosa-Pineda B, Chowdhury K, Torres M, Oliver G, Gruss P (1997) The Pax4 gene is essential for differentiation of insulin-producing bold beta cells in the mammalian pancreas. Nature 386: 399-402.

3. Ritz-Laser B, Estreicher A, Gauthier BR, Mamin A, Edlund H, et al. (2002) The pancreatic beta-cell-specific transcription factor Pax-4 inhibits glucagon gene expression through Pax-6. Diabetologia 45: 97-107.

4. Ding L, Yan J, Zhu J, Zhong H, Lu Q, et al. (2003) Ligand-independent activation of estrogen receptor alpha by XBP-1. Nucleic Acids Res 31: 5266-5274.

5. Laganière J, Deblois G, Lefebvre C, Bataille AR, Robert F, et al. (2005) From the Cover: Location analysis of estrogen receptor alpha target promoters reveals that FOXA1 defines a domain of the estrogen response. Proc Natl Acad Sci U S A 102: 11651-11656.

6. Madureira PA, Varshochi R, Constantinidou D, Francis RE, Coombes RC, et al. (2006) The Forkhead box M1 protein regulates the transcription of the estrogen receptor alpha in breast cancer cells. J Biol Chem 281: 25167-25176.

7. Zhang X, Leung YK, Ho SM (2007) AP-2 regulates the transcription of estrogen receptor (ER)-beta by acting through a methylation hotspot of the 0N promoter in prostate cancer cells. Oncogene 26: 7346-7354.

8. Lin JD, Liu C, S L (2008) Integration of energy metabolism and the mammalian clock. Cell Cycle 7: 453-457.

9. Zvonic S, Floyd ZE, Mynatt RL, Gimble JM (2007) Circadian rhythms and the regulation of metabolic tissue function and energy homeostasis. Obesity (Silver Spring) 15: 539-543.

10. Mitsui S, Yamaguchi S, Matsuo T, Ishida Y, Okamura H (2001) Antagonistic role of E4BP4 and PAR proteins in the circadian oscillatory mechanism. Genes Dev 15: 995-1006.

11. Hastings MH, Herzog ED (2004) Clock genes, oscillators, and cellular networks in the suprachiasmatic nuclei. J Biol Rhythms 19: 400-413.

12. Damiola F, Le Minh N, Preitner N, Kornmann B, Fleury-Olela F, et al. (2000) Restricted feeding uncouples circadian oscillators in peripheral tissues from the central pacemaker in the suprachiasmatic nucleus. Genes Dev 14: 2950-2961.

13. Hara R, Wan K, Wakamatsu H, Aida R, Moriya T, et al. (2007) Restricted feeding entrains liver clock without participation of the suprachiasmatic nucleus. Genes Cells 6: 269-278.

14. Yang X, Downes M, Yu RT, Bookout AL, He W, et al. (2006) Nuclear receptor expression links the circadian clock to metabolism. Cell 126: 801-810.

15. Krivan W, Wasserman WW (2001) A predictive model for regulatory sequences directing liver-specific transcription. Genome Res 11: 1559-1566.

16. Wasserman WW, Fickett JW (1998) Identification of regulatory regions which confer muscle-specific gene expression. J Mol Biol 278: 167-181.

17. Hastings M, O'Neill JS, Maywood ES (2007) Circadian clocks: regulators of endocrine and metabolic rhythms. J Endocrinol 195: 187-198.

18. Franklin AE, Engeland CG, Kavaliers M, Ossenkopp KP (2007) The rate of behavioral tolerance development to repeated lipopolysaccharide treatments depends upon the time of injection during the light-dark cycle: a multivariable examination of locomotor activity. Behav Brain Res 180: 161--173.

19. Gorbacheva VY, Kondratov RV, Zhang R, Cherukuri S, Gudkov AV, et al. (2005) Circadian sensitivity to the chemotherapeutic agent cyclophosphamide depends on the functional status of the CLOCK/BMAL1 transactivation complex. Proc Natl Acad Sci U S A 102: 3407-3412.

20. Ptitsyn AA, Gimble JM (2007) Analysis of circadian pattern reveals tissue-specific alternative transcription in leptin signaling pathway. BMC Bioinformatics 8: S15.
