# Supplementary material for: Regulation of Clock-Controlled Genes in Mammals
Source: PLoS One. 2009 Mar 16;4(3):e4882. doi: 10.1371/journal.pone.0004882 (PMC2654074; doi:10.1371/journal.pone.0004882)
Supplement: Text S3 — Additional references to the regulatory network presented in Figure 6. (0.08 MB DOC) [file pone.0004882.s006.doc]

**Supplement 5: Additional references to the regulatory network presented in Figure 6**

1. Alliston T, Ko TC, Cao Y, Liang YY, Feng XH, Chang C, Derynck R (2005) Repression of bone morphogenetic protein and activin-inducible transcription by Evi-1. *J Biol Chem.* **280**: 24227-37.
2. Andreucci JJ, Grant D, Cox DM, Tomc LK, Prywes R, Goldhamer DJ, Rodrigues N, Bédard PA, McDermott JC (2002) Composition and function of AP-1 transcription complexes during muscle cell differentiation.*J Biol Chem*. **277**:16426-32.
3. Biesiada E, Hamamori Y, Kedes L, Sartorelli V (1999) Myogenic Basic Helix-Loop-Helix Proteins and Sp1 Interact as Components of a Multiprotein Transcriptional Complex Required for Activity of the Human Cardiac á-Actin Promoter. *Mol Cell Biol*. **19**: 2577–2584.
4. Blokzijl A, ten Dijke P, Ibáñez C (2003) Physical and Functional Interaction between GATA-3 and Smad3 Allows TGF-â Regulation of GATA Target Genes. *Current Biology* **12:** 35-45
5. Blyszczuk P, Czyz J, Kania G, Wagner M, Roll U, St-Onge L, Wobus AM (2003) Expression of Pax4 in embryonic stem cells promotes differentiation of nestin-positive progenitor and insulin-producing cells. *Proc Natl Acad Sci U S A*. **100**: 998-1003.
6. Boudreau F, Rings EH, van Wering HM, Kim RK, Swain GP, Krasinski SD, Moffett J, Grand RJ, Suh ER, Traber PG (2002) Hepatocyte nuclear factor-1 alpha, GATA-4, and caudal related homeodomain protein Cdx2 interact functionally to modulate intestinal gene transcription. Implication for the developmental regulation of the sucrase-isomaltase gene. *J Biol Chem*. **277**: 31909-17.
7. Brand-Saberi B (2005) Genetic and epigenetic control of skeletal muscle development. *Ann Anat.* **187**: 199-207.
8. Brown CO 3rd, Chi X, Garcia-Gras E, Shirai M, Feng XH, Schwartz RJ (2004) The cardiac determination factor, Nkx2-5, is activated by mutual cofactors GATA-4 and Smad1/4 via a novel upstream enhancer. *J Biol Chem*. **279**: 10659-69.
9. Brown M., Goldstein J (1997) The SREBP Pathway: Regulation of Cholesterol Metabolism by Proteolysis of a Membrane-Bound Transcription Factor. *Cell* **89:** 331-340
10. Buchberger, A., K. Ragge, and H. H. Arnold. 1994. The myogenin gene is activated during myocyte differentiation by pre-existing, not newly synthesized transcription factor MEF-2. *J. Biol. Chem.* **269**: 17289-17296.

Ma K, Chan JK, Zhu G, Wu Z (2005) Myocyte enhancer factor 2 acetylation by p300 enhances its DNA binding activity, transcriptional activity, and myogenic differentiation. *Mol Cell Biol.* **25**:3575-82.

Molkentin, J. D., B. L. Black, J. F. Martin, and E. N. Olson (1995) Cooperative activation of muscle gene expression by MEF2 and myogenic bHLH proteins. *Cell* **83**: 1125-1136.

1. Buser AC, Gass-Handel EK, Wyszomierski SL, Doppler W, Leonhardt SA, Schaack J, Rosen JM, Watkin H, Anderson SM, Edwards DP (2007) Progesterone receptor repression of prolactin/signal transducer and activator of transcription 5-mediated transcription of the beta-casein gene in mammary epithelial cells. *Mol Endocrinol*.**21**:106-25.

Rocha-Viegas L, Vicent GP, Barañao JL, Beato M, Pecci A (2006) Glucocorticoids repress bcl-X expression in lymphoid cells by recruiting STAT5B to the P4 promoter.*J Biol Chem*. **281**: 33959-70.

1. Cai W, Rambaud J, Teboul M, Masse I, Benoit G, Gustafsson JA, Delaunay F, Laudet V, Pongratz I (2008) Expression levels of estrogen receptor beta are modulated by components of the molecular clock. *Mol Cell Biol.* **28**: 784-93.
2. Cavadini G, Petrzilka S, Kohler P, Jud C, Tobler I, Birchler T, Fontana A (2007) TNF-alpha suppresses the expression of clock genes by interfering with E-box-mediated transcription. *Proc Natl Acad Sci U S A.* **104**: 12843-8.
3. Chakravarti D, LaMorte VJ, Nelson MC, Nakajima T, Schulman IG, Juguilon H, Montminy M, Evans RM (1996) Role of CBP/P300 in nuclear receptor signalling *Nature* **383**: 99-103.
4. Chilov D, Hofer T, Bauer C, Wenger RH, Gassmann M (2001) Hypoxia affects expression of circadian genes PER1 and CLOCK in mouse brain. *FASEB J*. **15**: 2613-22.
5. Claudel T, Cretenet G, Saumet A, Gachon F (2007) Crosstalk between xenobiotics metabolism and circadian clock. *FEBS Lett.* **581**: 3626-33.

Garrett RW, Gasiewicz TA (2006) The aryl hydrocarbon receptor agonist 2,3,7,8-tetrachlorodibenzo-p-dioxin alters the circadian rhythms, quiescence, and expression of clock genes in murine hematopoietic stem and progenitor cells. *Mol. Pharmacol.* **69**: 2076-83.

Mukai M, Tischkau SA (2007) Effects of tryptophan photoproducts in the circadian timing system: searching for a physiological role for aryl hydrocarbon receptor. *Toxicol Sci*. **95**: 172-81.

1. De Luca A, Severino A, De Paolis P, Cottone G, De Luca L, De Falco M, Porcellini A, Volpe M, Condorelli G (2003) p300/cAMP-response-element-binding-protein ('CREB')-binding protein (CBP) modulates co-operation between myocyte enhancer factor 2A (MEF2A) and thyroid hormone receptor-retinoid X receptor. *Biochem J.* **369**: 477-84.
2. Desaint S, Hansmannel F, Clémencet MC, Le Jossic-Corcos C, Nicolas-Frances V, Latruffe N, Cherkaoui-Malki M (2004) NFY interacts with the promoter region of two genes involved in the rat peroxisomal fatty acid beta-oxidation: the multifunctional protein type 1 and the 3-ketoacyl-CoA B thiolase. *Lipids Health Dis.* **3**:4.
3. Duez H, Staels B (2008) Rev-erb alpha gives a time cue to metabolism. *FEBS Lett*. **582**: 19-25.

Laitinen S, Fontaine C, Fruchart JC, Staels B (2005) The role of the orphan nuclear receptor Rev-Erb alpha in adipocyte differentiation and function. *Biochimie* **87**: 21-5.

1. Ema M, Hirota K, Mimura J, Abe H, Yodoi J, Sogawa K, Poellinger L, Fujii-Kuriyama Y (1999)Molecular mechanisms of transcription activation by HLF and HIF1alpha in response to hypoxia: their stabilization and redox signal-induced interaction with CBP/p300.*EMBO J*. **18**: 1905-14.
2. Espinosa L, Inglés-Esteve J, Robert-Moreno A, Bigas A (2003) IkappaBalpha and p65 regulate the cytoplasmic shuttling of nuclear corepressors: cross-talk between Notch and NFkappaB pathways. *Mol Biol Cell*. **14**: 491-502.
3. Etchegaray JP, Lee C, Wade PA, Reppert SM (2003) Rhythmic histone acetylation underlies transcription in the mammalian circadian clock. *Nature* **421**: 177-82.
4. Ezoe S, Matsumura I, Gale K, Satoh Y, Ishikawa J, Mizuki M, Takahashi S, Minegishi N, Nakajima K, Yamamoto M, Enver T, Kanakura Y (2005) GATA transcription factors inhibit cytokine-dependent growth and survival of a hematopoietic cell line through the inhibition of STAT3 activity. *J. Biol. Chem.* **280**:13163–13170.

Huang Z, Richmond TD, Muntean AG, Barber DL, Weiss MJ, Crispino JD (2007) STAT1 promotes megakaryopoiesis downstream of GATA-1 in mice. *J Clin Invest*. **117**: 3890-9.

1. Fang Y, Yan J, Ding L, Liu Y, Zhu J, Huang C, Zhao H, Lu Q, Zhang X, Yang X, Ye Q (2004) XBP-1 increases ERalpha transcriptional activity through regulation of large-scale chromatin unfolding. *Biochem Biophys Res Commun.* **323**: 269-74.
2. Foti D, Iuliano R, Chiefari E, Brunetti A.(2003) A nucleoprotein complex containing Sp1, C/EBP beta, and HMGI-Y controls human insulin receptor gene transcription. *Mol Cell Biol.* 23(8):2720-32.
3. Fraser DJ, Zumsteg A, Meyer UA. (2003) Nuclear receptors constitutive androstane receptor and pregnane X receptor activate a drug-responsive enhancer of the murine 5-aminolevulinic acid synthase gene.*J Biol Chem.* **278**: 39392-401.

May BK, Dogra SC, Sadlon TJ, Bhasker CR, Cox TC, Bottomley SS (1995) Molecular regulation of heme biosynthesis in higher vertebrates.*Prog Nucleic Acid Res Mol Biol*.**51**: 1-51

1. Gachon F, Olela FF, Schaad O, Descombes P, Schibler U (2006) The circadian PAR-domain basic leucine zipper transcription factors DBP, TEF, and HLF modulate basal and inducible xenobiotic detoxification. *Cell Metab*. **4**: 25-36.
2. Galon J, Sudarshan C, Ito S, Finbloom D, O'Shea JJ (1999) IL-12 induces IFN regulating factor-1 (IRF-1) gene expression in human NK and T cells. *J Immunol.* **162**: 7256-62.

Lehtonen A, Lund R, Lahesmaa R, Julkunen I, Sareneva T, Matikainen S (2003) IFN-alpha and IL-12 activate IFN regulatory factor 1 (IRF-1), IRF-4, and IRF-8 gene expression in human NK and T cells. *Cytokine* **24**: 81-90.

1. Gekakis N, Staknis D, Nguyen HB, Davis FC, Wilsbacher LD, King DP, Takahashi JS, Weitz CJ (1998) Role of the CLOCK protein in the mammalian circadian mechanism. *Science* **280**: 1564-9.

Shearman LP, Jin X, Lee C, Reppert SM, Weaver DR (2000) Targeted disruption of the mPer3 gene: subtle effects on circadian clock function. *Mol Cell Biol*. **20**: 6269-75.

1. Ghilardi N, Ziegler S, Wiestner A, Stoffel R, Heim MH, Skoda RC (1996) Defective STAT signaling by the leptin receptor in diabetic mice. *Proc Natl Acad Sci U S A.* **93**: 6231-5.
2. Gonzalez MI, Robins DM (2001) Oct-1 preferentially interacts with androgen receptor in a DNA-dependent manner that facilitates recruitment of SRC-1. *J Biol Chem*. **276**: 6420-8.

Préfontaine GG, Walther R, Giffin W, Lemieux ME, Pope L, Haché RJ (1999) Selective binding of steroid hormone receptors to octamer transcription factors determines transcriptional synergism at the mouse mammary tumor virus promoter. *J Biol Chem.* **274:** 26713-9.

1. Görlach A, Bonello S (2008) The cross-talk between NF-kappaB and HIF-1: further evidence for a significant liaison. *Biochem J.* **412**:e17-9.
2. Gregory RC, Taxman DJ, Seshasayee D, Kensinger MH, Bieker JJ, Wojchowski DM (1996)Functional interaction of GATA1 with erythroid Krüppel-like factor and Sp1 at defined erythroid promoters. *Blood*. **87**:1793-801.
3. Guillaumond F, Dardente H, Giguère V, Cermakian N (2005) Differential control of Bmal1 circadian transcription by REV-ERB and ROR nuclear receptors. *J Biol Rhythms* **20**: 391-403.
4. Hanlon M, Sturgill TW, Sealy L (2001) ERK2- and p90Rsk2-dependent Pathways Regulate the CCAAT/Enhancer-binding Protein- Interaction with Serum Response Factor. *J Biol Chem.* **276**: 38449-56.
5. Harroch S, Revel M, Chebath J (1994) Induction by interleukin-6 of interferon regulatory factor 1 (IRF-1) gene expression through the palindromic interferon response element pIRE and cell type-dependent control of IRF-1 binding to DNA. *EMBO J.* **13**: 1942-9.
6. Hashimoto K, Matsumoto S, Yamada M, Satoh T, Mori M (2007)Liver X receptor-alpha gene expression is positively regulated by thyroid hormone. *Endocrinology* **148**: 4667-75.
7. Hastings M, O'Niel JS, Maywood ES (2007) Circadian clocks: regulators of endocrine and metabolic rhythms. *Journal of Endocrinology* **195**: 187-198.
8. He H, Soncin F, Grammatikakis N, Li Y, Siganou A, Gong J, Brown SA, Kingston RE, Calderwood SK (2003) Elevated expression of heat shock factor (HSF) 2A stimulates HSF1-induced transcription during stress. *J Biol Chem.* **278**: 35465-75
9. Hirano F, Tanaka H, Hirano Y, Hiramoto M, Handa H, Makino I, Scheidereit C (1998) Functional Interference of Sp1 and NF-kappa B through the Same DNA Binding Site. *Mol Cell Biol* **18**: 1266-1274.

Lee K, Burgoon LD, Lamb L, Dere E, Zacharewski TR, Hogenesch JB, LaPres JJ (2006) Identification and characterization of genes susceptible to transcriptional cross-talk between the hypoxia and dioxin signaling cascades. *Chem Res Toxicol.* **19**: 1284-93.

1. Hirose T, Sowa Y, Takahashi S, Saito S, Yasuda C, Shindo N, Furuichi K, Sakai T. (2003) p53-independent induction of Gadd45 by histone deacetylase inhibitor: coordinate regulation by transcription factors Oct-1 and NF-Y.*Oncogene*. **22**: 7762-73.

Peng Y, Jahroudi N (2002) The NFY transcription factor functions as a repressor and activator of the von Willebrand factor promoter. *Blood* **99**: 2408-17.

Schwachtgen JL, Remacle JE, Janel N, Brys R, Huylebroeck D, Meyer D, Kerbiriou-Nabias D (1998) Oct-1 Is Involved in the Transcriptional Repression of the von Willebrand Factor Gene Promoter. *Blood* **92:** 1247-1258.

1. Hirota T, Okano T, Kokame K, Shirotani-Ikejima H, Miyata T, Fukada Y (2002) Glucose down-regulates Per1 and Per2 mRNA levels and induces circadian gene expression in cultured Rat-1 fibroblasts. *J Biol Chem*. **277**: 44244-51.
2. Hung HC, Maurer C, Kay SA, Weber F (2007) Circadian transcription depends on limiting amounts of the transcription co-activator nejire/CBP. *J Biol Chem.* **282**: 31349-57.
3. Inouye S, Fujimoto M, Nakamura T, Takaki E, Hayashida N, Hai T, Nakai A (2007) Heat shock transcription factor 1 opens chromatin structure of interleukin-6 promoter to facilitate binding of an activator or a repressor. *J Biol Chem*. **282**: 33210-7.
4. Jensen J. (2004) Gene regulatory factors in pancreatic development.*Dev Dyn*. **229**:176-200.
5. Kaplan J, Calame K (1997) The ZiN/POZ domain of ZF5 is required for both transcriptional activation and repression. *Nucleic Acids Res*. **25**: 1108-16.
6. Kim HS, Kim MS, Hancock AL, Harper JCP, Park JY, Poy G, Perantoni AO, Cam M, Malik K, Lee SB (2007) Identification of novel WT1 target genes implicated in kidney development. *J Biol Chem*. **282**: 16278-87
7. Kim JW, Monila H, Pandey A, Lane MD (2007) Upstream stimulatory factors regulate the C/EBP alpha gene during differentiation of 3T3-L1 preadipocytes. *Biochem Biophys Res Commun.* **354**: 517-21.
8. Kim SK, Selleri L, Lee JS, Zhang AY, Gu X, Jacobs Y, Cleary ML (2002) Pbx1 inactivation disrupts pancreas development and in Ipf1-deficient mice promotes diabetes mellitus. *Nat Genet.* **30**: 430-5.
9. Kokkonen N, Ulibarri IF, Kauppila A, Luosujärvi H, Rivinoja A, Pospiech H, Kellokumpu I, Kellokumpu S (2007) Hypoxia upregulates carcinoembryonic antigen expression in cancer cells.Int J Cancer. **121**: 2443-50.
10. Levi F, Schibler U (2007) Circadian rhythms: mechanisms and therapeutic implications.*Annu Rev Pharmacol Toxicol*. **47**: 593-628.
11. Li Y, Song X, Ma Y, Liu J, Yang D, Yan B (2004) DNA binding, but not interaction with Bmal1, is responsible for DEC1-mediated transcription regulation of the circadian gene mPer1. *Biochem J*. **382**: 895–904
12. Lin SC (2006) Identification of an NF-Y/HMG-I(Y)-binding site in the human IL-10 promoter. *Mol Immunol.* **43**: 1325-31.
13. Lin WY, Hu YJ, Lee YH (2008) Hepatocyte Nuclear Factor-1a Regulates Glucocorticoid Receptor Expression to Control Postnatal Body Growth.*Am J Physiol Gastrointest Liver Physiol.* doi:10.1152/ajpgi.00081.2008
14. Liu X, Popescu IR, Denisova JV, Neve RL, Corriveau RA, Belousov AB (2008) Regulation of Cholinergic Phenotype in Developing Neurons. *J Neurophysiol*. **23**: 2680-98.

Tian X, Feig LA (2006) Age-dependent participation of Ras-GRF proteins in coupling calcium-permeable AMPA glutamate receptors to Ras/Erk signaling in cortical neurons. *J Biol Chem*. **281**: 7578-82.

1. McPherson LA, Weigel RJ (1999) AP2alpha and AP2gamma: a comparison of binding site specificity and trans-activation of the estrogen receptor promoter and single site promoter constructs. *Nucleic Acids Res*. **27**: 4040-9.
2. Miki N, Ikuta M, Matsui T (2004) Hypoxia-induced activation of the retinoic acid receptor-related orphan receptor alpha4 gene by an interaction between hypoxia-inducible factor-1 and Sp1. *J Biol Chem*. **279**: 15025-31.
3. Motzkus D, Albrecht U, Maronde E (2002) The human PER1 gene is inducible by interleukin-6. *J Mol Neurosci.* **18**: 105-9.
4. Myatt SS, Lam EW-F (2007) The emerging roles of forkhead box (FOX) proteins in cancer. *Nature* **7**: 847-859
5. Numata A, Shimoda K, Kamezaki K, Haro T, Kakumitsu H, Shide K, Kato K, Miyamoto T, Yamashita Y, Oshima Y, Nakajima H, Iwama A, Aoki K, Takase K, Gondo H, Mano H, Harada M (2005) Signal transducers and activators of transcription 3 augments the transcriptional activity of CCAAT/enhancer-binding protein alpha in granulocyte colony-stimulating factor signaling pathway. *J Biol Chem.* **280**: 12621-9.
6. Oberg F, Wu S, Bahram F, Nilsson K, Larsson LG (2001) Cytokine-induced restoration of differentiation and cell cycle arrest in v-Myc transformed U-937 monoblasts correlates with reduced Myc activity. *Leukemia* **15**: 217-27.
7. Ohdo S, Koyanagi S, Suyama H, Higuchi S, Aramaki H (2001) Changing the dosing schedule minimizes the disruptive effects of interferon on clock function. *Nat Med*. **7**: 356-60.
8. Ohlsson H, Karlsson K, Edlund T (1993) IPF1, a homeodomain-containing transactivator of the insulin gene. *EMBO J*. **12**: 4251-9.
9. Ohno T, Onishi Y, Ishida N (2007) A novel E4BP4 element drives circadian expression of mPeriod2. *Nucleic Acids Res*. **35**: 648-55.
10. Piantadosi CA, Suliman HB (2008) Transcriptional Regulation of SDHa flavoprotein by nuclear respiratory factor-1 prevents pseudo-hypoxia in aerobic cardiac cells. *J Biol Chem.* **283**: 10967-77.
11. Preitner N, Damiola F, Lopez-Molina L, Zakany J, Duboule D, Albrecht U, Schibler U (2002) The orphan nuclear receptor REV-ERBalpha controls circadian transcription within the positive limb of the mammalian circadian oscillator. *Cell* **110**: 251-60.
12. Pulivarthy SR, Tanaka N, Welsh DK, De Haro L, Verma IM, Panda S (2007) Reciprocity between phase shifts and amplitude changes in the mammalian circadian clock. *Proc Natl Acad Sci* **104**: 20356-61.
13. Qadri I, Iwahashi M, Kullak-Ublick GA, Simon FR (2006) Hepatocyte nuclear factor (HNF) 1 and HNF4 mediate hepatic multidrug resistance protein 2 up-regulation during hepatitis C virus gene expression. *Mol Pharmacol.* **70**: 627-36.
14. Radoja N, Komine M, Jho SH, Blumenberg M, Tomic-Canic M (2000) Novel mechanism of steroid action in skin through glucocorticoid receptor monomers. *Mol Cell Biol.* **20**: 4328-39.
15. Raghuram S, Stayrook KR, Huang P, Rogers PM, Nosie AK, McClure DB, Burris LL, Khorasanizadeh S, Burris TP, Rastinejad F (2007) Identification of heme as the ligand for the orphan nuclear receptors REV-ERBalpha and REV-ERBbeta. *Nat Struct Mol Biol*.**14**: 1207-13.
16. Ramsauer K, Farlik M, Zupkovitz G, Seiser C, Kröger A, Hauser H, Decker T (2007) Distinct modes of action applied by transcription factors STAT1 and IRF1 to initiate transcription of the IFN-gamma-inducible gbp2 gene. *Proc Natl Acad Sci U S A*. **104**: 2849-54.

Tang X, Gao JS, Guan YJ, McLane KE, Yuan ZL, Ramratnam B, Chin YE. (2007) Acetylation-dependent signal transduction for type I interferon receptor. *Cell* **131**: 93-105.

1. Rathinam C, Klein C. (2007) Transcriptional repressor Gfi1 integrates cytokine-receptor signals controlling B-cell differentiation.*PLoS ONE* **2**: e306.
2. Ripperger JA, Schibler U (2006) Rhythmic CLOCK-BMAL1 binding to multiple E-box motifs drives circadian Dbp transcription and chromatin transitions. *Nat Genet.* **38**: 369-74.
3. Roder K, Zhang L, Schweizer M (2007) SREBP-1c mediates the retinoid-dependent increase in fatty acid synthase promoter activity in HepG2. *FEBS Lett*. **581**: 2715-20.
4. Roth U, Curth K, Unterman TG, Kietzmann T (2003) The transcription factors HIF-1 and HNF-4 and the coactivator p300 are involved in insulin-regulated glucokinase gene expression via the phosphatidylinositol 3-kinase/protein kinase B pathway. *J Biol Chem*. **279**: 2623-31.
5. Saadane N, Alpert L, Chalifour LE. (2000) Altered molecular response to adrenoreceptor-induced cardiac hypertrophy in Egr-1-deficient mice. *Am J Physiol Heart Circ Physiol*. **278**: 796-805.
6. Sánchez-Elsner T, Ramírez JR, Sanz-Rodriguez F, Varela E, Bernabéu C, Botella LM. (2004) A cross-talk between hypoxia and TGF-beta orchestrates erythropoietin gene regulation through SP1 and Smads. *J Mol Biol*. **336**:9-24.
7. Santalucía T, Moreno H, Palacín M, Yacoub MH, Brand NJ, Zorzano A (2001) A novel functional co-operation between MyoD, MEF2 and TRalpha1 is sufficient for the induction of GLUT4 gene transcription. *J Mol Biol.* **314**: 195-204.
8. Sirito M, Lin Q, Maity T, Sawadogo M (1994) Ubiquitous expression of the 43- and 44-kDa forms of transcription factor USF in mammalian cells.*Nucleic Acids Res*. **22**:427-33.
9. Slattery C, Ryan MP, McMorrow T. (2008) E2A proteins: regulators of cell phenotype in normal physiology and disease.*Int J Biochem Cell Biol.* **40**: 1431-6.
10. Smith S, Watada H, Scheel D, Mrejen C, German M (2000) Autoregulation and maturity onset diabetes of the young transcription factors control the human PAX4 promoter. *J. Biol. Chem.* **275:** 36910?36919.
11. Solomon DL, Amati B, Land H (1993) Distinct DNA binding preferences for the c-Myc/Max and Max/Max dimers. *Nucleic Acids Res*.**21**: 5372-6.
12. Sonoda J, Laganière J, Mehl IR, Barish GD, Chong LW, Li X, Scheffler IE, Mock DC, Bataille AR, Robert F, Lee CH, Giguère V, Evans RM (2007) Nuclear receptor ERR alpha and coactivator PGC-1 beta are effectors of IFN-gamma-induced host defense. *Genes Dev*. **21**: 1909-20.
13. Suh JH, Gong EY, Kim JB, Lee IK, Choi HS, Lee K (2008) Sterol regulatory element-binding protein-1c represses the transactivation of androgen receptor and androgen-dependent growth of prostatic cells. *Mol Cancer Res.* **6**: 314-24.
14. Sun L, Trausch-Azar JS, Muglia LJ, Schwartz AL (2008) Glucocorticoids differentially regulate degradation of MyoD and Id1 by N-terminal ubiquitination to promote muscle protein catabolism.*Proc Natl Acad Sci U S A*. **105**: 3339-44.
15. Teboul M, Guillaumond F, Gréchez-Cassiau A, Delaunay F. (2008) The Nuclear Hormone Receptors Family Round the Clock. *Mol Endocrinol*. Jul 24.[Epub ahead of print]
16. Tremblay JJ, Hamel F, Viger RS (2002) Protein kinase A-dependent cooperation between GATA and CCAAT/enhancer-binding protein transcription factors regulates steroidogenic acute regulatory protein promoter activity.*Endocrinology* **143**: 3935-45.
17. Ueda HR, Chen W, Adachi A, Wakamatsu H, Hayashi S, Takasugi T, Nagano M, Nakahama K, Suzuki Y, Sugano S, Iino M, Shigeyoshi Y, Hashimoto S (2002) A transcription factor response element for gene expression during circadian night. *Nature* **418**: 534-9.
18. Wada T, Kang HS, Angers M, Gong H, Bhatia S, Khadem S, Ren S, Ellis E, Strom SC, Jetten AM, Xie W (2008) Identification of oxysterol 7alpha-hydroxylase (Cyp7b1) as a novel retinoid-related orphan receptor alpha (RORalpha) (NR1F1) target gene and a functional cross-talk between RORalpha and liver X receptor (NR1H3). *Mol Pharmacol*. **73**: 891-9.
19. Wang H, Larris B, Peiris TH, Zhang L, Le Lay J, Gao Y, Greenbaum LE (2007) C/EBPbeta activates E2F-regulated genes in vivo via recruitment of the coactivator CREB-binding protein/P300. *J Biol Chem*. **282**: 24679-88.
20. Wang N, Kim HG, Cotta CV, Wan M, Tang Y, Klug CA, Cao X (2006) TGFbeta/BMP inhibits the bone marrow transformation capability of Hoxa9 by repressing its DNA-binding ability. *EMBO Journal* **25**: 1469-80.
21. Yamamoto T, Shimano H, Nakagawa Y, Ide T, Yahagi N, Matsuzaka T, Nakakuki M, Takahashi A, Suzuki H, Sone H, Toyoshima H, Sato R, Yamada N (2004) SREBP-1 interacts with hepatocyte nuclear factor-4 alpha and interferes with PGC-1 recruitment to suppress hepatic gluconeogenic genes. *J Biol Chem.* **279**:12027-35.
22. Yu X, Zhu X, Pi W, Ling J, Ko L, Takeda Y, Tuan D (2005) The long terminal repeat (LTR) of ERV-9 human endogenous retrovirus binds to NF-Y in the assembly of an active LTR enhancer complex NF-Y/MZF1/GATA-2. *J Biol Chem*. **280:** 35184-35194.
23. Zada AA, Pulikkan JA, Bararia D, Geletu M, Trivedi AK, Balkhi MY, Hiddemann WD, Tenen DG, Behre HM, Behre G. (2006)Proteomic discovery of Max as a novel interacting partner of C/EBPalpha: a Myc/Max/Mad link.*Leukemia*. **20**: 2137-46.
24. Zhang F, Lin M, Abidi P, Thiel G, Liu J (2003) Specific interaction of Egr1 and c/EBPbeta leads to the transcriptional activation of the human low density lipoprotein receptor gene. *J Biol Chem.* **278**: 44246-54.
25. Zhang X, Azhar G, Zhong Y, Wei JY (2004) Identification of a novel serum response factor cofactor in cardiac gene regulation. *J Biol Chem.* **279**: 55626-32.
26. Zhang X, Liu Y (2003) Suppression of HGF receptor gene expression by oxidative stress is mediated through the interplay between Sp1 and Egr-1. *Am J Physiol Renal Physiol*. **284**: 1216-25.
27. Zhao J, Kong HJ, Li H, Huang B, Yang M, Zhu C, Bogunovic M, Zheng F, Mayer L, Ozato K, Jay Unkeless, Xiong H (2006) JIRF-8/Interferon (IFN) Consensus Sequence-binding Protein Is Involved in Toll-like Receptor (TLR) Signaling and Contributes to the Cross-talk between TLR and IFN-{gamma} Signaling Pathways. *J. Biol. Chem*. **281:** 10073-10080.
